# Insecticide resistance and cytochrome-P450 activation in unfed and blood-fed laboratory and field populations of *Culex pipiens pallens*

**DOI:** 10.1007/s10340-016-0820-1

**Published:** 2016-12-03

**Authors:** Kyu-Sik Chang, Heung-Chul Kim, Terry A. Klein, Young Ran Ju

**Affiliations:** 1 National Institute of Health, Korea Center for Disease Control and Prevention, Cheongju-si, Chungbuk 28159 Republic of Korea; 2 5th Medical Detachment, 168th Multifunctional Medical Battalion, 65th Medical Brigade, Unit 15247, APO, AP 96205-5247 USA; 3 Medical Department Activity Korea, 65th Medical Brigade, Unit 15281, APO, AP 96205-5281 USA

**Keywords:** *Culex pipiens pallens*, Resistance, Insecticide, Esterases, Detoxification, Cytochrome, P450, Glutathione *S*-transferases

## Abstract

Understanding the mechanisms of insecticide resistance to vector mosquitoes is critical for the implementation of effective control measures. A nulliparous susceptible *Culex pipiens pallens* (KSCP) laboratory colony and two field strains from Paju (PAJ) and Jeonju (JEO) Korea were evaluated for susceptibility to five pesticides by microapplication techniques. Unfed PAJ and JEO females demonstrated increased resistance compared to unfed KSCP females, respectively. While blood-fed KSCP females demonstrated <10-fold decreased susceptibility to pesticides compared to unfed KSCP females, blood-fed PAJ and JEO females demonstrated 25.0–50.0- and 16.0–38.6-fold increased resistance compared to unfed PAJ and JEO females, respectively. Unfed and blood-fed groups were assayed for α- and β-esterase, glutathione *S*-transferases, and cytochrome P-450 (P450) enzyme activity assays. P450 activity was 58.8- and 72.8-fold higher for unfed PAJ and JEO females, respectively, than unfed KSCP females. P450 enzyme activity of KSCP females assayed 1 and 7 days after a blood meal increased by 14.5- and 11.8-fold, respectively, compared to unfed KSCP females, while PAJ and JEO females demonstrated 164.9- and 148.5- and 170.7- and 160.4-fold increased activity, respectively, compared to unfed females of each population. However, other three resistance-related metabolic enzymes showed low activation at <10-fold after a blood meal. The data demonstrate that P450 acts on elevated insecticide resistance after blood meals in resistant field populations. Our findings might reveal that suppressing of the P450 protein by artificial gene mutation increases insecticidal susceptibility of *Cx*. *pipiens* and will promise effective vector mosquito control.

## Key message

- Understanding mechanisms of insecticide resistance among mosquito vectors of pathogens affecting human health is critical for the implementation of effective control measures.
- Data show that the P450 enzyme is a major factor for increased insecticide resistance of field populations of *Culex pipiens*, including synergistic effects with esterases and glutathione *S*-transferase.
- Mosquitoes provided with blood meals demonstrated increased activity of P450 and increased insecticide resistance.
- Management of the activation of the P450 enzyme in *Cx*. *pipiens* may provide insights into effective control.

## Introduction

The common house mosquito, *Culex pipiens pallens* Coquillett, is a nuisance biting mosquito in the Republic of Korea (ROK) (Kim et al. 2007a, b; Shin et al. 2012). In the United States and China, it is a vector of West Nile virus (WNV) (Anonymous 2007; Jiang et al. 2014). Distribution of the mosquitoes is strongly influenced by the availability of human-generated habitat similar to *Aedes albopictus*, a dengue virus vector (Heersink et al. 2015), and under urban conditions population density is very high. In some Asian countries, it is a primary vector of epidemic encephalitis and *Wucheraria bancrofti* and *Brugia malayi*, the causative agents for lymphatic filariasis (Ye 1995; Rowland et al. 1999). Of immediate concern is the introduction of WNV to the ROK based on the potential transport of virus-infected mosquitoes via daily airline arrivals from the US and other endemic countries (Tsuda 2005). To address these concerns, in 2010, the Korea Centers for Disease Control and Prevention (KCDC) and the Japan National Institute of Infectious Disease (JNIID) specified WN encephalitis and dengue fever as potential national epidemics due to the high risks associated with the introduction of infected mosquitoes into the ROK and Japan (KCDC 2014; JNIID 2015). Integrated pest management, including biological and mechanical control, is used for the control of mosquitoes and associated pathogens affecting human and veterinary health. However, when these methods fail or are impractical to implement, chemical control measures are often instituted. To ensure that effective integrated pest management measures are instituted, including chemical control, factors that affect the susceptibility of vector populations to registered insecticides approved for larval and adult mosquito control in the ROK should be evaluated.

Insecticide resistance is based on enhanced enzymatic sequestration and detoxification of active ingredients and metabolites of insecticides, as well as the alteration of insecticide target sites leading to insecticide insensitivity. Improved enzymatic detoxification has been linked to three broad classes of enzymes, e.g., cytochrome P450 monooxygenase (P450), glutathione *S*-transferase (GST), and non-specific hydrolases (e.g., α-esterase and β-esterase) (Terriere 1984; Hemingway 2000; Hemingway et al. 2004; Spilling et al. 2008; Cuamba et al. 2010).

Many primary biological processes affect the activation of insecticide detoxification enzymes other than insecticide exposure. While blood meals serve as a source of protein for egg development, studies also show that blood-fed adult females demonstrated enhanced insecticide resistance when compared to unfed nulliparous females. For example, an insecticide-susceptible *Anopheles funestus* strain of unfed females did not demonstrate significant differences in susceptibility to pyrethroid insecticides, while blood-fed females demonstrated decreased susceptibility (Spilling et al. 2008). These data suggest that insecticide detoxification mechanisms involved in insecticide resistance are stimulated following a blood meal, leading to enhanced expression of the resistance phenotype.

Here, we report the relative decreased susceptibility/resistance levels of a *Cx. p. pallens* susceptible laboratory colony and two field-collected strains from Paju (PAJ) and Jeonju (JEO) in the Republic of Korea to selected pesticides, and observed changes in the quantity of four metabolic detoxification enzymes before and after blood feeding.

## Materials and methods

### Laboratory colony

A laboratory strain of *Cx*. *p*. *pallens* (KSCP) susceptible to organophosphate and pyrethroid insecticides originated from Tongilchon, Baegyeon-ri, Gunnae-myeon, Paju-si, Gyeonggi Province, ROK, and maintained by the Division of Entomology, Korea National Institute of Health (KNIH), for more than 10 years without exposure to insecticides was used as the control group. Adult mosquitoes were provided with a 10% sucrose solution and maintained at 27 ± 2 °C, 65–75% relative humidity (RH), and 12:12 h light:dark cycle. For egg development, 1- to 3-day-old females were provided with blood meals on white laboratory mice which were placed in a secure screened cage to restrict movement for up to 1 h, under a Korea National Institute of Health Institutional Animal Care and Use Committee (KCDC-020-11-2A) protocol approved for this study.

Fully engorged females were placed in a screened cage and eggs removed daily and placed in plastic larval-rearing pans (27 × 15 × 4 cm) containing 500 ml dechlorinated water. After hatching, larvae were separated into groups of approximately 200 larvae/rearing pan and provided with 0.5 g of sterilized diet daily (Vivid-S : Super TetraMin^®^; 4:1 ratio by weight) (Sewhapet, Incheon, ROK). Pupae were transferred daily to emergence cups containing water to 3 cm depth, and then placed in 0.5-m^3^ screened cages and provided with a 10% sucrose solution as a food source for emerging adults and maintained as described above.

### Field test strains

**Fig. 1:**
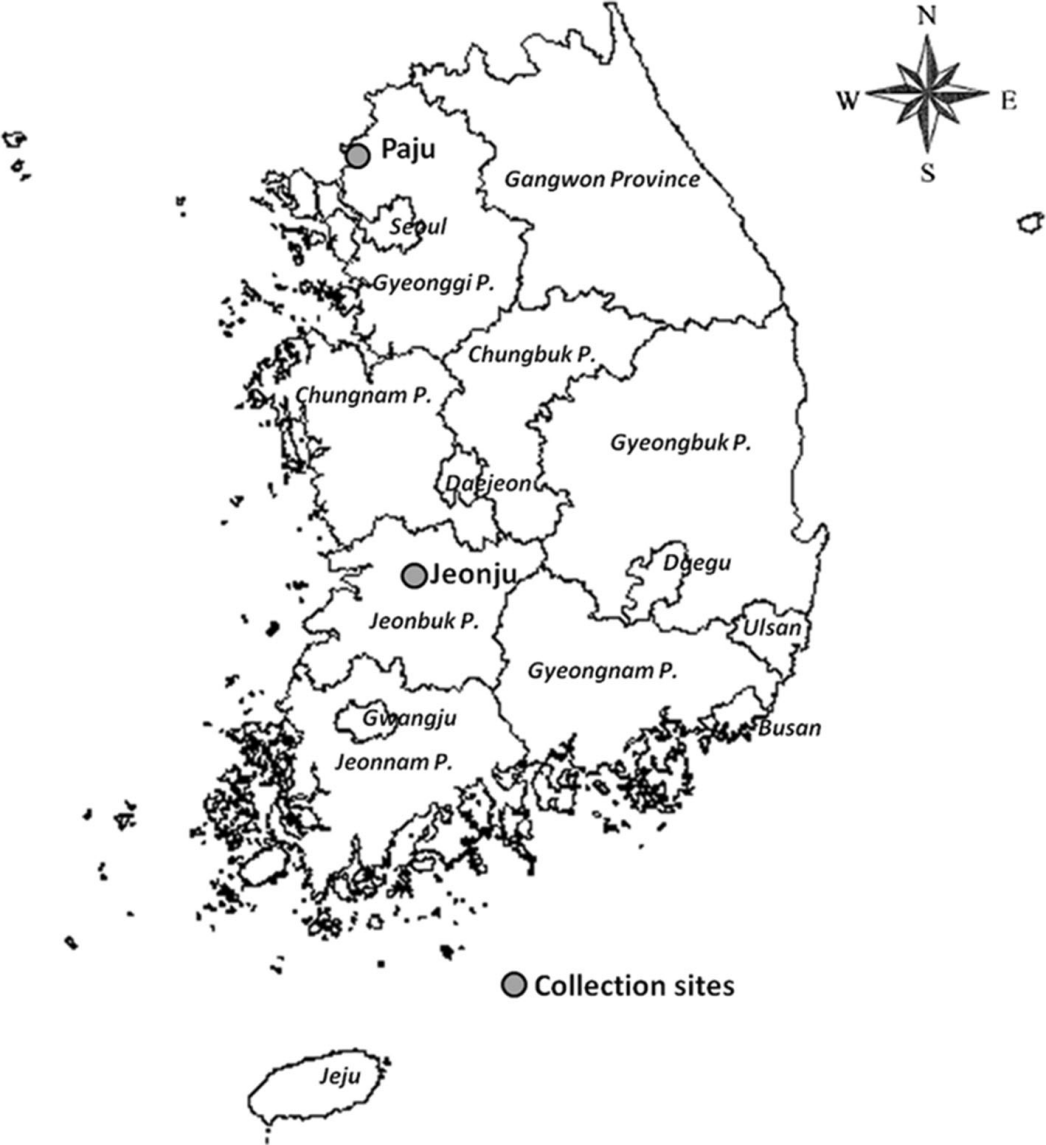
Field mosquito collection sites. The map was generated using ArcGis for Desktop (Esri, v.10.3.1.4959)

Two collection sites of *Culex p*. *pallens* are shown in Fig. 1 (ESRI 2015).

*Culex p*. *pallens* larvae were collected from organic pools of water near rice paddies and cow sheds at Paju (37°54′37·34″N, 126°43·37′12″E), Gyeonggi Province (Paju strain, PAJ) and at Jeonju (35°48′14·31″N, 127°11′36·73″E), Jeollabuk Province (Jeonju strain, JEO) from June to September 2012 after receiving approval (oral) from the farm owners and under a KNIH protocol approved for this study. Field-collected larvae were returned to the mosquito-rearing facility at KNIH and similarly reared to adults (*F*_0_) as described above. A total of 2–4 specimens from each collection site were assayed by PCR for species confirmation (Kasai et al. 2008). Approximately 250 1- to 3-day-old *F*_0_ females were provided with blood meals on white laboratory mice for egg development, and eggs were obtained and the larvae reared to adults (*F*_1_) as described above.

### Bioassay

#### Topical application

A total of 25 1- to 3-day-old females from each strain from the KSCP susceptible laboratory strain and *F*_1_ progeny of the field test strains (PAJ, and JEO) were separated into three groups consisting of (1) unengorged nulliparous females provided with a 10% sucrose solution until assayed on day 7 (U7), (2) fully engorged females assayed on day 1 (E1) post-blood feeding, and (3) fully engorged females assayed on day 7 (E7) post-blood feeding. For the blood-fed groups, the sucrose solution was removed and 1- to 3-day-old unfed females were placed in 500-cm^3^ screen-topped cartons (7 cm high × 9.5 cm deep) 4 h prior to allow them to blood feed directly on a cow for 15 min in accordance with an approved protocol under good laboratory practices and after receiving approval (oral) from the farm owner. After blood feeding, fully engorged mosquitoes were separated, and partially and non-engorged mosquitoes were discarded in accordance with the standard protocol. Engorged females were maintained separately from males and were not provided the an oviposition substrate.

Insecticides [bifenthrin (97.0% purity), deltamethrin (99.5%), chlorpyrifos (98.5%), fenthion (95.5%), and fenitrothion (98.5%)] used for topical application were obtained from Fluka™ (Buchs, Switzerland). A total of 25 females from each group [unengorged + 7 days (U7), engorged + 1 day (E1), and engorged + 7 days (E7)] and strain (KSCP, PAJ, and JEO strains) were tested using 3–7 concentrations of each insecticide to obtain LC_50_ values and replicated three times. After females were briefly anaesthetized with ether on an ice plate (Fryka-Kältetechnik, Esslingen, Germany), 0.1 μl of insecticide dissolved in reagent grade solvent (acetone) in concentrations that resulted in 10–90% mortality were applied to the pronotum of each mosquito by a microapplicator (Burkard, Ricksmanworth, UK) and replicated 3–4 times as outlined by the World Health Organization (WHO 2006). After application, mosquitoes were placed in 500-cm^3^ screen-topped cartons, provided with a 10% sucrose solution (saturated cotton) on the screened top and held for 24 h at 27 ± 2 °C and 60 ± 10% RH. At the end of 24 h, dead and moribund mosquitoes were counted and the data recorded. Resistant ratios (RR) for each of the strains tested were defined as the LC_50_ value for each blood-fed (E1 and E7) group/LC_50_ values of the unfed (U7) group for each of the insecticides.

### Microplate enzyme assays

#### Enzymes and reagents

Microplate enzyme assays on all enzyme activation were assayed according to the method of Brogdon (2010) with minor modifications following Methods in *Anopheles* research of US CDC.

Enzymes and reagents used for microplate enzyme assays, e.g., dithiobis (2-nitrobenzoic acid) (DTNB), potassium phosphate buffer (KPO_4_ buffer), α-naphthyl, β-naphthyl, cytochrome-C, *ο*-dianisidine tetrazotized (Practical Grade, D3), sodium acetate (NaOAc) buffer, bovine serum albumin (BSA), reduced glutathione, glacial acetic acid, 3,3′,5,5′-tetramethyl-benzidine dihydrochloride (TMBZ), and hydrogen peroxide (H_2_O_2_), were purchased from Sigma-Aldrich^®^ (St. Louis, MO, USA). Acetocholine levels were not determined as they are not related to blood meals.

#### Preparation for microplate enzyme activity assays

A total of 100 each of unfed and blood-fed *Cx*. *p*. *pallens* females from each of the three groups (1- to 3-day-old unfed females provided with a 10% sucrose solution and assayed on day 7 and 1- to 3-day-old blood-fed females assayed on days 1 and 7 post-blood feeding) for each of the “control” (KSCP) and “field test” (PAJ and JEO) strains were killed by placing them in an ultra-low temperature freezer (−20 °C) for 10 min. The specimens were then removed and homogenized individually on ice in 1.5-ml microcentrifuge tubes with 100 μl of KPO_4_ buffer using glass beads (Daihan Scientific, Seoul, Korea). The homogenates were spun at 14,000*g* for 2 min at 4 °C in an ultracentrifuge and the supernatant used as a crude enzyme extract for biochemical assays. Ninety-six wells microplates were used for quantification of the four enzymes and total protein. A total of three replicates of 100 female mosquitoes each, including a minimum of three positive and three negative controls, were assayed for cytochrome P450 monooxygenase (P450), glutathione *S*-transferase (GST), non-specific hydrolase (α- and β-esterase), and total protein. Total protein was corrected for size variation among the specimens (Brogdon 1984) using a Bradford (Coomassie) protein assay kit (Gendepot, TX, USA) according to the user’s guide. A total of 100 μl of each standard or unknown sample and 150 μl of the Bradford reagent were added to each well and then mixed on a plate shaker for 30 s. After mixing, the microplates were removed and incubated for 10 min at room temperature. The absorbance (optical density, OD) values for each of the samples were measured using a kinetic microplate reader (Molecular Devices, CA, USA) at 595 nm. Mean measurements of the blank replicates were subtracted from individual measurements of the standard and mosquito samples. A standard curve and formula were drawn using Microsoft Excel 2011 to determine total protein concentrations for each of the samples by plotting the mean values at 595 nm of the blank corrected measurements for each bovine serum albumin (BSA) standard versus its concentration in μg/ml. The mean protein (μg/100 μl homogenate) for each mosquito sample was measured three times and the mean protein and the volume (μl) corresponding to 4 μg of protein was calculated for each mosquito before being used in the enzyme biochemical assays. A total of 4 μg protein (by volume) for each of the mosquito samples was added to each well for the enzyme assays as described below.

#### Microplate glutathione *S*-transferase (GST) assay

The GST activity was assayed according to the method of Brogdon (2010) with minor modifications following Methods in *Anopheles* research of US CDC. A total of 61 mg of reduced glutathione was dissolved in 100 ml of KPO_4_ buffer and stored at 4 °C for 3–4 days. A total of 20 mg of 1-chloro-2,4-dinitrobenzene (cDNB) was dissolved in 10 ml of acetone, followed by adding 90 ml of 0.25 M KPO_4_ buffer. A total of 100 μl of KPO_4_ buffer was added to 36 wells containing 4 μg protein of mosquito homogenate for the laboratory control KSCP (10) and field-collected test PAJ (10) and JEO (10) groups and negative (3) and positive (3) controls, followed by adding 100 μl of reduced glutathione solution and cDNB solution to each well. OD values were immediately read at 340 nm using a kinetic microplate reader (Molecular Devices) (*T*_0_) and reread at 5 min (*T*_5_). OD values calculated as: OD = *T*_0_ − *T*_5_.

#### Microplate non-specific hydrolase (EST, α and β esterase) assay

α-Esterase and β-esterase activity was determined according to the method of Brogdon (2010) with minor modifications following Methods in *Anopheles* research of US CDC. A total of 100 μl of KPO_4_ buffer was added to 36 wells containing 4 μg of mosquito sample homogenates for the control KSCP (10) and test PAJ (10) and JEO (10) groups and negative (3) and positive (3) controls, followed by 100 μl of α- or β-naphthol and then incubated at room temperature for 10 min. A total of 100 mg of *ο*-dianisidine tetrazotized was dissolved in 100 ml of purified water immediately before use, and then 100 μl of *ο*-dianisidine solution was added to each well and incubated for 2 min at room temperature. OD values were immediately read at 620 and 540 nm to determine α-esterase and β-esterase activity, respectively.

#### Microplate cytochrome P450 monooxygenase assay

Mosquito homogenates were assayed to determine cytochrome P450 activity according to the method of Brogdon (2010) with minor modifications following Methods in *Anopheles* research of US CDC. NaOAc buffer consisted of 0.25 M sodium acetate (C_2_H_2_NaO_2_) dissolved in 800 ml purified water and then adjusted to pH 5.0 with acetic acid. A total of 20 mg of TMBZ was dissolved in 25 ml absolute methanol and then 75 ml of 0.25 M NaOAc buffer (pH 5.0) added. The TMBZ solution was stored at 4 °C and discarded after 3 days, or before if it turned a light blue color.

A total of 10 mg of cytochrome-C was added to 100 ml of 0.25 M NaOAc buffer (pH 5.0) and the final product used as the oxidase positive control stock. A total of 100 μl of KPO_4_ buffer was added to 36 wells containing 4 μg protein from mosquito homogenates of the control KSCP (10) and test PAJ (10) and JEO (10) groups and negative (3) and positive (3) controls. A total of 100 μl of the cytochrome-C positive control solution was added to three wells for positive controls, while 200 μl of TMBZ solution was added to each test well, followed by adding one drop (≈25 μl) of 3% hydrogen peroxide (H_2_O_2_). After incubation for 5 min at room temperature, OD values were read at 620 nm and oxidase activity determined.

### Data analysis

Insecticide concentrations and mortality data were subjected to probit analysis (SAS 2004). The LC_50_ values for each treatment were considered to be significantly different at 95% confidence limits. Resistance ratios (RR) for the comparison of unfed and blood-fed groups were calculated as: RR = LC_50_ of blood-fed females for each of the group strains/LC_50_ of unfed nulliparous females of corresponding strains.

Reaction rates (Rr) were calculated as the enzyme activity value for unfed and blood-fed females from each of the strains and groups/unfed 7-day-old nulliparous females (KSCP-U7). Rr values of <10, 10–40, 40–160, and >160 were characterized as low, moderate, high, and extremely high resistance, respectively (Kim et al. 2004).

The Bonferroni multiple-comparison method was used to test for significant differences of activation of metabolic detoxification enzymes in unfed nulliparous and blood-fed *Culex pipiens pallens* females from a susceptible laboratory colony (KSCP), and field-collected strains from Paju, Gyeonggi Province (PAJ strain) and Jeonju, Jeollabuk Province (JEO strain) (SAS 9.13 program, 2nd edn; SAS Institute, Cary, NC, USA). Means ± standard error (SE) of untransformed data are reported.

## Results

### Topical-application assay

**Fig. 2:**
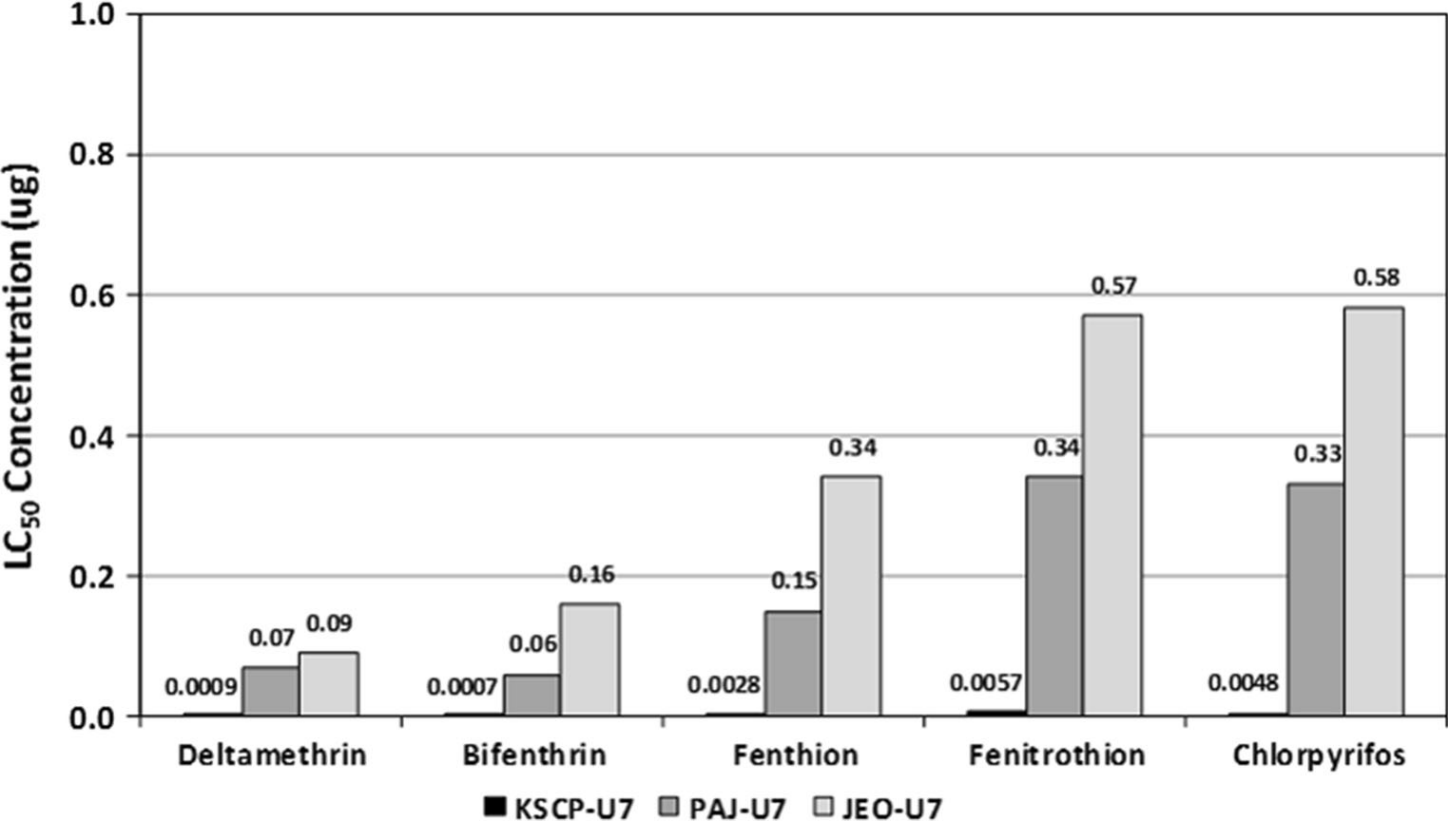
LC_50_ values (µg/♀) and proportional decreased susceptibility/resistance of 1- to 3-day-old unfed nulliparous female *Culex pipiens pallens* (provided with 10% sucrose solution and held for 7 days) for KSCP susceptible laboratory strain (control), Paju (PAJ), and Jeonju (JEO) stains exposed to five insecticides

**Fig. 3:**
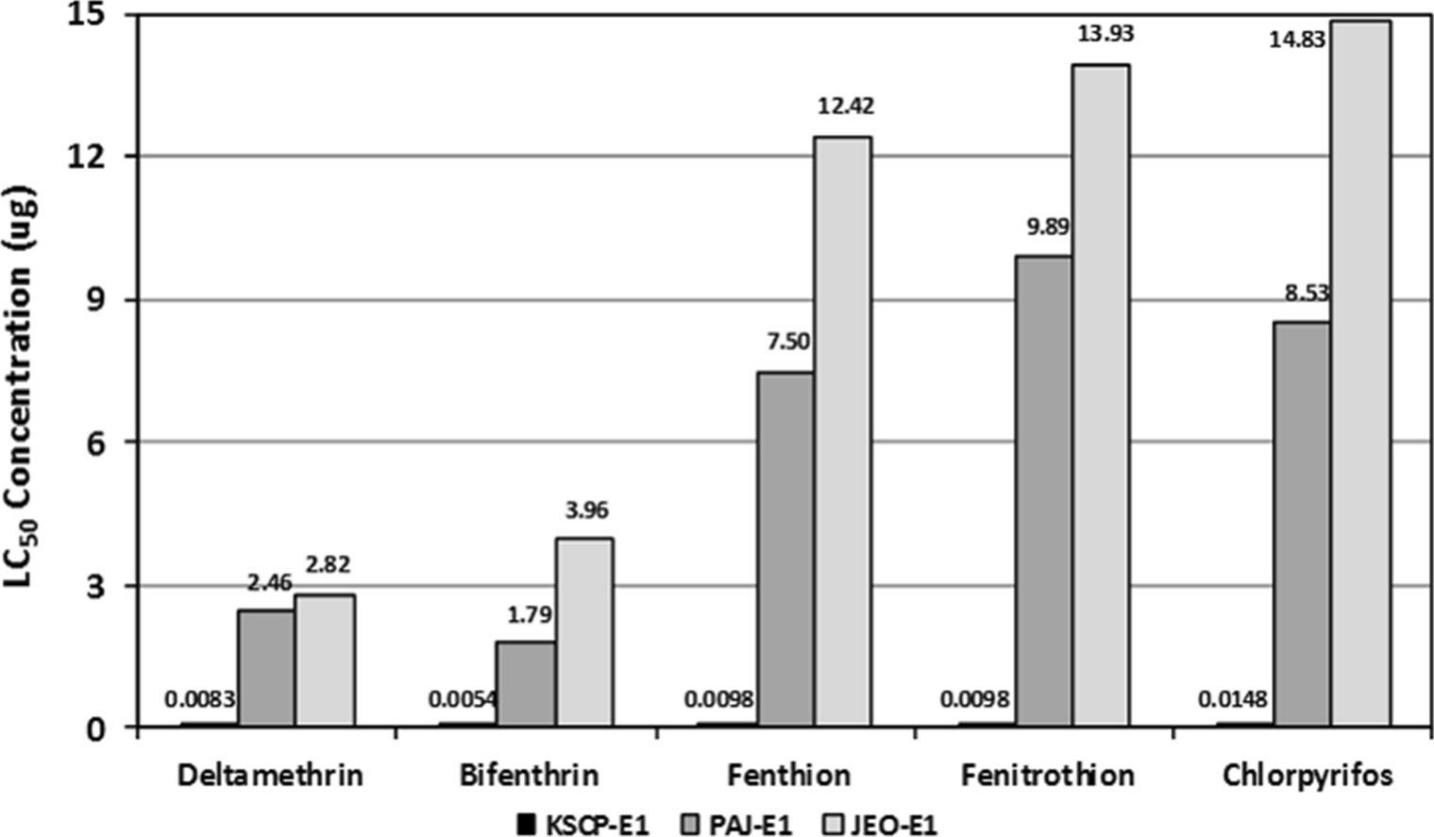
LC_50_ values (µg/♀) and proportional increased decreased susceptibility/resistance of 1- to 3-day-old blood-fed *Culex pipiens pallens* females for KSCP susceptible laboratory strain (control), Paju (PAJ), and Jeonju (JEO) stains exposed to five insecticides 1 day after blood feeding

**Fig. 4:**
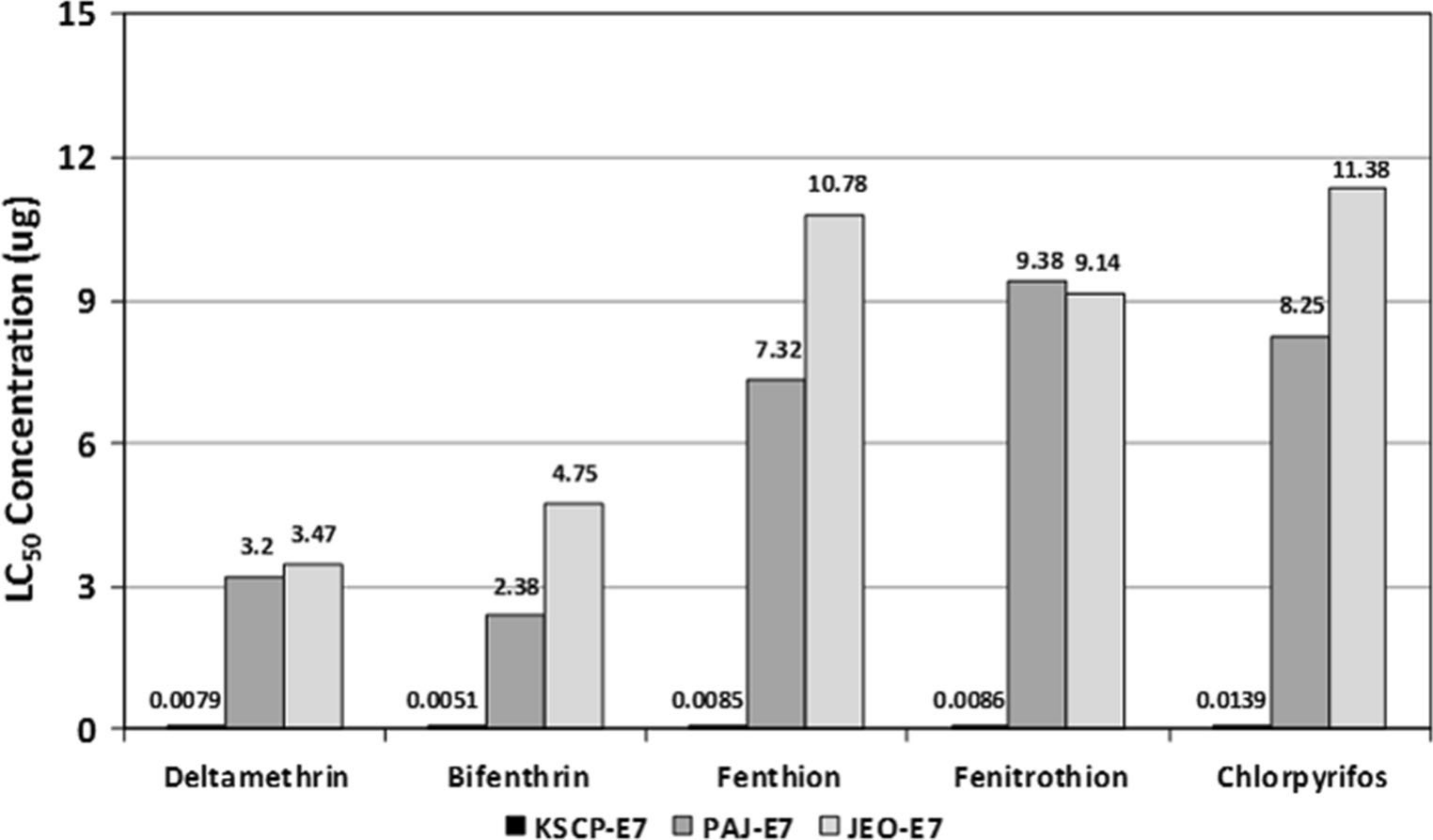
LC_50_ values (µg/♀) and proportional increased decreased susceptibility/resistance of 1- to 3-day-old blood-fed *Culex pipiens pallens* females for KSCP susceptible laboratory strain (control), Paju (PAJ), and Jeonju (JEO) stains exposed to five insecticides 7 days after blood feeding

**Fig. 5:**
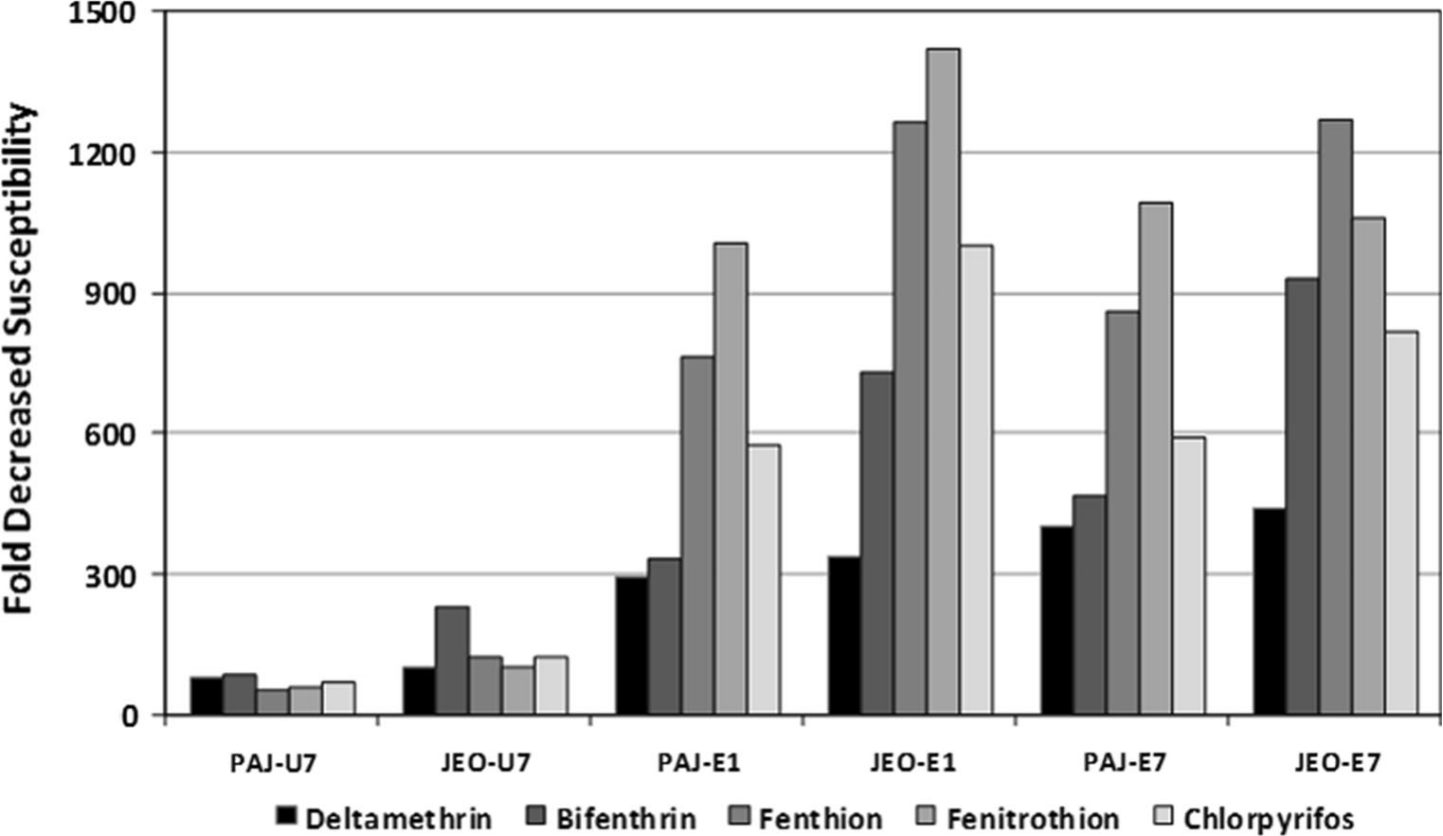
The proportional increased decreased susceptibility/resistance for 1- to 3-day-old *Culex pipiens pallens* Paju (PAJ) and Jeonju (JEO) stains of unfed nulliparous females assayed 7 days following feeding on a 10% sucrose solution and engorged females assayed 1 and 7 days after blood feeding compared to KSCP (susceptible laboratory strain) unfed and blood-fed (engorged) females

Unfed 7 day-old (U7) females demonstrated lower LC_50_ (µg/♀) values than blood-fed females assayed on days 1 (E1) and 7 (E7) post-blood feeding for all insecticides tested (Table 1; Figs. 2, 3, 4). These data indicate an increased decreased susceptibility/resistance among blood-fed females shortly after a blood meal (E1) and after complete digestion of the blood meal (E7). The KSCP strain demonstrated the least differences in insecticide decreased susceptibility (<10-fold difference) with resistance ratios ranging from a low of 1.5 (KSCP-E7) for fenitrothion to a high of 9.2 (KSCP-E1) for deltamethrin (Table 1; Fig. 2). PAJ-E1 and PAJ-E7) blood-fed females demonstrated a high degree of resistance (>10-fold difference) to all insecticides tested when compared to the unfed (PAJ-U7) females, ranging from a low of 25.0 (PAJ-E7) for chlorpyrifos to a high of 50.0 (PAJ-E1) for fenthion (Tables 1; Fig. 3). Similarly, JEO-E1 and JEO-E7 blood-fed females demonstrated a high degree of resistance to all insecticides tested when compared to the unfed (JEO-U7) females, ranging from a low of 16.0 (JEO-E7) for fenitrothion to a high of 38.6 (JEO-E7) for deltamethrin (Table 1; Fig. 4). Both the PAJ-U7 and JEO-U7 groups demonstrated similar levels of decreased susceptibility/resistance to each insecticide when compared to the KSCP-U7 group (Fig. 5). Increased resistance levels were similar for all of the PAJ-E1/E7 and JEO-E1/E7 blood-fed groups for each of the insecticides when compared with the corresponding KSCP-E1/E7 blood-fed groups. In general, the lowest level of resistance was observed for the PAJ-E1/E7 and JEO-E1/E7 blood-fed groups to deltamethrin, while the highest levels of resistance were observed for fenitrothion and fenthion when compared to corresponding KSCP-E1/E7 blood-fed groups.

**Table 1:** Comparative toxicity after 24 h exposure to five insecticides applied to the prothorax of unfed (non-blood-fed) and blood-fed (engorged) female *Culex pipiens pallens* laboratory strain (*KSCP*) and field-collected strains (*PAJ* and* JEO*) using a micro-application assay

Insecticide	Strain/group^a,b,c^	*n* ^d^	Slope ± SE	LC_50_ (µg/♀, CL^e^)	*χ* ^2^	RR^f^
Bifenthrin	KSCP-U7	375	2.7 ± 0.91	0.0007 (0.0006–0.0008)	3.32	1
	PAJ-U7	300	1.0 ± 0.14	0.06 (0.04–0.08)	0.43	85.7
	JEO-U7	450	0.9 ± 0.08	0.16 (0.11–0.22)	6.38	228.6
	KSCP-E1	450	0.5 ± 0.05	0.0054 (0.0041–0.0062)	6.67	7.7
	PAJ-E1	450	1.3 ± 0.09	1.79 (1.34–2.41)	6.67	29.8
	JEO-E1	375	1.3 ± 0.15	3.96 (3.03–5.39)	5.52	24.8
	KSCP-E7	450	0.6 ± 0.05	0.0051 (0.0031–0.0067)	2.97	7.3
	PAJ-E7	375	1.0 ± 0.11	2.38 (1.71–3.13)	2.97	39.7
	JEO-E7	450	1.2 ± 0.12	4.75 (3.58–5.98)	2.09	29.7
Deltamethrin	KSCP-U7	450	2.5 ± 0.62	0.0009 (0.0007–0.0011)	2.7	1
	PAJ-U7	375	1.2 ± 0.12	0.07 (0.05–0.09)	4.2	77.8
	JEO-U7	450	1.1 ± 0.10	0.09 (0.07–0.13)	6.14	100.0
	KSCP-E1	450	0.6 ± 0.05	0.0083 (0.0072–0.0103)	1.31	9.2
	PAJ-E1	450	1.1 ± 0.09	2.46 (1.84–3.41)	1.31	35.1
	JEO-E1	375	1.4 ± 0.13	2.82 (2.19–3.71)	4.03	31.3
	KSCP-E7	375	1.2 ± 0.11	0.0079 (0.0058–0.0089)	3.58	8.8
	PAJ-E7	375	1.1 ± 0.12	3.20 (2.35–4.60)	3.58	45.7
	JEO-E7	450	1.1 ± 0.10	3.47 (2.53–4.68)	1.62	38.6
Fenthion	KSCP-U7	375	1.0 ± 0.11	0.0028 (0.0020–0.0038)	4.77	1
	PAJ-U7	375	1.3 ± 0.12	0.15 (0.12–0.20)	6.19	53.6
	JEO-U7	450	0.8 ± 0.08	0.34 (0.24–0.49)	3.77	121.4
	KSCP-E1	375	0.9 ± 0.10	0.0098 (0.0087–0.0139)	3.52	3.5
	PAJ-E1	375	1.2 ± 0.13	7.50 (5.72–9.10)	3.52	50
	JEO-E1	375	2.0 ± 0.25	12.42 (10.55–14.78)	5.11	36.5
	KSCP-E7	375	1.1 ± 0.11	0.0085 (0.0078–0.0098)	1.36	3
	PAJ-E7	375	1.3 ± 0.14	7.32 (5.68–8.21)	1.36	48.8
	JEO-E7	375	2.0 ± 0.24	10.78 (9.14–11.32)	4.89	31.7
Chlorpyrifos	KSCP-U7	450	1.0 ± 0.09	0.0048 (0.0036–0.0061)	6.66	1
	PAJ-U7	375	1.3 ± 0.13	0.33 (0.25–0.45)	0.82	68.8
	JEO-U7	450	0.9 ± 0.09	0.58 (0.41–0.69)	6.02	120.8
	KSCP-E1	375	1.2 ± 0.12	0.0148 (0.0139–0.0238)	2.33	3.1
	PAJ-E1	375	1.6 ± 0.16	8.53 (7.81–9.69)	2.33	25.8
	JEO-E1	375	1.9 ± 0.26	14.83 (12.48–18.61)	1.16	25.6
	KSCP-E7	375	1.0 ± 0.11	0.0139 (0.0102–0.0191)	4.08	2.9
	PAJ-E7	375	1.2 ± 0.13	8.25 (7.49–9.30)	4.09	25
	JEO-E7	375	1.9 ± 0.21	11.38 (10.85–13.65)	4.07	19.6
Fenitrothion	KSCP-U7	450	1.1 ± 0.09	0.0057 (0.0049–0.0073)	4.25	1
	PAJ-U7	375	1.1 ± 0.12	0.34 (0.25–0.48)	6.06	59.6
	JEO-U7	450	0.9 ± 0.08	0.57 (0.44–0.61)	7.17	100.0
	KSCP-E1	450	0.8 ± 0.08	0.0098 (0.0080–0.0140)	4.88	1.7
	PAJ-E1	450	1.8 ± 0.16	9.89 (8.61–10.76)	4.88	29.1
	JEO-E1	375	2.0 ± 0.26	13.93 (11.75–14.60)	0.73	24.4
	KSCP-E7	375	1.1 ± 0.13	0.0086 (0.0072–0.0116)	0.69	1.5
	PAJ-E7	375	1.9 ± 0.15	9.38 (7.93–11.29)	0.69	27.6
	JEO-E7	375	1.3 ± 0.15	9.14 (8.21–10.72)	3.1	16

^a^KSCP-U7; PAJ-U7; JEO-U7, 1- to 3-day-old female *Cx*. *p*. *pallens*, susceptible laboratory strain and two field strains (PAJ and JEO), provided with a 10% sugar solution (unfed) for 7 days prior to micro-application of selected pesticides

^b^KSCP-E1; PAJ-E1; JEO-E1, 1- to 3-day-old female *Cx*. *p*. *pallens*, susceptible laboratory strain and two field strains (PAJ and JEO), provided with bovine blood from cows (engorged) and held for 1 day prior to micro-application of selected pesticides

^c^KSCP-E7; PAJ-E7; JEO-E7, 1- to 3-day-old female *Cx*. *p*. *pallens*, susceptible laboratory strain and two field strains (PAJ and JEO), provided with bovine blood from cows and held for 7 days prior to micro-application of selected pesticides

^d^Mosquito number tested

^e^Confidence limits

^f^ *RR* relative susceptibility; PAJ-U7: each insecticide LC_50_ PAJ-U7/corresponding insecticide LC_50_ KSCP-U7; JEO-U7: each insecticide LC_50_ JEO-U7/corresponding insecticide LC_50_ KSCP-U7; KSCP-E1: each insecticide LC_50_ KSCP-E1/corresponding insecticide LC_50_ KSCP-U7; KSCP-E7: each insecticide LC_50_ KSCP-E7/corresponding insecticide LC_50_ KSCP-U7

The slopes for KSCP-U7 groups treated with pyrethroids (bifenthrin and deltamethrin, 2.7 ± 0.91 and 2.5 ± 0.62, respectively) were much greater when compared to KSCP-E1 (0.5 ± 0.05 and 0.6 ± 0.05, respectively) and KSCP-E7 (0.6 ± 0.05 and 1.2 ± 0.05, respectively), whereas the slope of KSCP-U7, E1 and E7 groups treated with organophosphates (fenthion, chlorpyrifos, and fenitrothion) were similar (Table 1). The slopes of PAJ-U7, E1, and E7 and JEO-U7, E1, and E7 for all pesticides were similar (Table 1).

### Micro plate enzyme assays

The activation of four metabolic detoxification enzymes, α-esterase, β-esterase, GST, and P450 was evaluated using the microplate enzyme test for unfed and blood-fed KSCP, PAJ, and JEO strain females (Table 2). For the laboratory (control) strain, the KSCP-U7 group demonstrated the highest levels for β-esterase (0.626 nmol/mg/min), followed by α-esterase (0.134 nmol/mg/min), GST (0.034 nmol/mg/min), and P450 (0.008 nmol/mg/min). Increased quantities of β-esterase and α-esterase for KSCP-E1 and KSCP-E7 blood-fed females were similar, ranging from 1.4 to 1.3 and 1.1 to 0.8, respectively. The quantities of GST increased 3.8–5.3-fold for KSCP-E1 and KSCP-E7 females, respectively, while the quantity of P450 increased >10-fold for both groups.

**Table 2:** Activation of metabolic detoxification enzymes in unfed nulliparous and blood-fed *Culex pipiens pallens* females from a susceptible laboratory colony (*KSCP*), and field-collected strains from Paju, Gyeonggi Province (*PAJ* strain) and Jeonju, Jeollabuk Province (*JEO* strain)

Test strain^a^	Enzyme^b^	Substrates	Enzyme (nmol/mg/min, mean ± SE)^c^					
			U7^d^	RrU7^e^	E1^d^	RrE1^f^	E7^d^	RrE7^g^
KSCP	α-E^h^	1-NA	0.134 ± 0.46ab	–	0.174 ± 0.23a	1.3	0.107 ± 0.16b	0.8
PAJ	α-E	1-NA	2.184 ± 0.34b	16.8	2.856 ± 0.12a	1.3	2.944 ± 0.17a	1.3
JEO	α-E	1-NA	2.472 ± 0.06c	18.4	3.838 ± 0.07b	1.6	4.252 ± 0.06a	1.7
KSCP	β-E^h^	2-NA	0.626 ± 0.45b	–	0.876 ± 0.40a	1.4	0.689 ± 0.35b	1.1
PAJ	β-E	2-NA	4.890 ± 0.48b	7.8	6.445 ± 0.26b	1.3	7.306 ± 0.40a	1.5
JEO	β-E	2-NA	6.118 ± 0.07c	9.8	7.146 ± 0.05b	1.2	8.564 ± 0.07a	1.4
KSCP	GST^h^	CDNB	0.034 ± 0.26b	–	0.129 ± 0.01a	3.8	0.180 ± 0.02a	5.3
PAJ	GST	CDNB	0.583 ± 0.05b	17.1	2.318 ± 0.30b	4	5.477 ± 0.50a	9.4
JEO	GST	CDNB	1.042 ± 0.05c	30.6	4.793 ± 0.07b	4.6	8.348 ± 0.06a	8
KSCP	P450^h^	TMBZ	0.008 ± 0.04c	–	0.116 ± 0.01a	14.5	0.094 ± 0.09b	11.8
PAJ	P450	TMBZ	0.470 ± 0.08c	58.8	77.485 ± 4.03a	164.9	69.782 ± 0.18b	148.5
JEO	P450	TMBZ	0.582 ± 0.04c	72.8	99.326 ± 0.29a	170.7	93.378 ± 0.92b	160.4

^a^KSCP: KNIH susceptible laboratory strain; PAJ: field-collected (larvae) from Paju, Gyeonggi Province; JEO: field-collected (larvae) from Jeonju, Jeollabuk Province

^b^α-E: α-esterase, β-E: β-esterase, GST: glutathione *S*-transferases, and P450: cytochrome P 450 monooxygenase

^c^Means within each column followed by the same letter are not significantly different (*P* = 0.05; Bonferroni method)

^d^U7: unfed 1–3-day old adult female *Cx. p. pallens* provided with a 10% sucrose solution and assayed for enzyme activity at 7-days; E1: 1- to 3-day-old adult female *Cx. p. pallens* assayed for enzyme activity on day 1 after a blood meal; E7: 1–33 day-old adult female *Cx*. *p*. *pallens* assayed for enzyme activity on day 7 after a blood meal

^e^RrU7: enzyme amount of PAJ-U7 or JEO-U7/enzyme amount of KSCP-U7

^f^RrE1: enzyme amount of PAJ-E1 or JEO-E1/enzyme amount of PAJ-U1 or JEO-U1

^g^RrE7: enzyme amount of PAJ-E7 or JEO-E7/enzyme amount of PAJ-U7 or JEO-U7

^h^α-E *P* values for KSCP, PAJ and JEO strains: 0.0445, 0.017 and <0.0001, respectively; β-E *P* values for KSCP, PAJ and JEO strains: 0.001, <0.001 and <0.0001, respectively; GST *P* values for KSCP, PAJ and JEO strains: <0.001, <0.001 and <0.0001, respectively; P450 *P* values for KSCP, PAJ and JEO strains: <0.001, <0.0001 and <0.0001, respectively

**Fig. 6:**
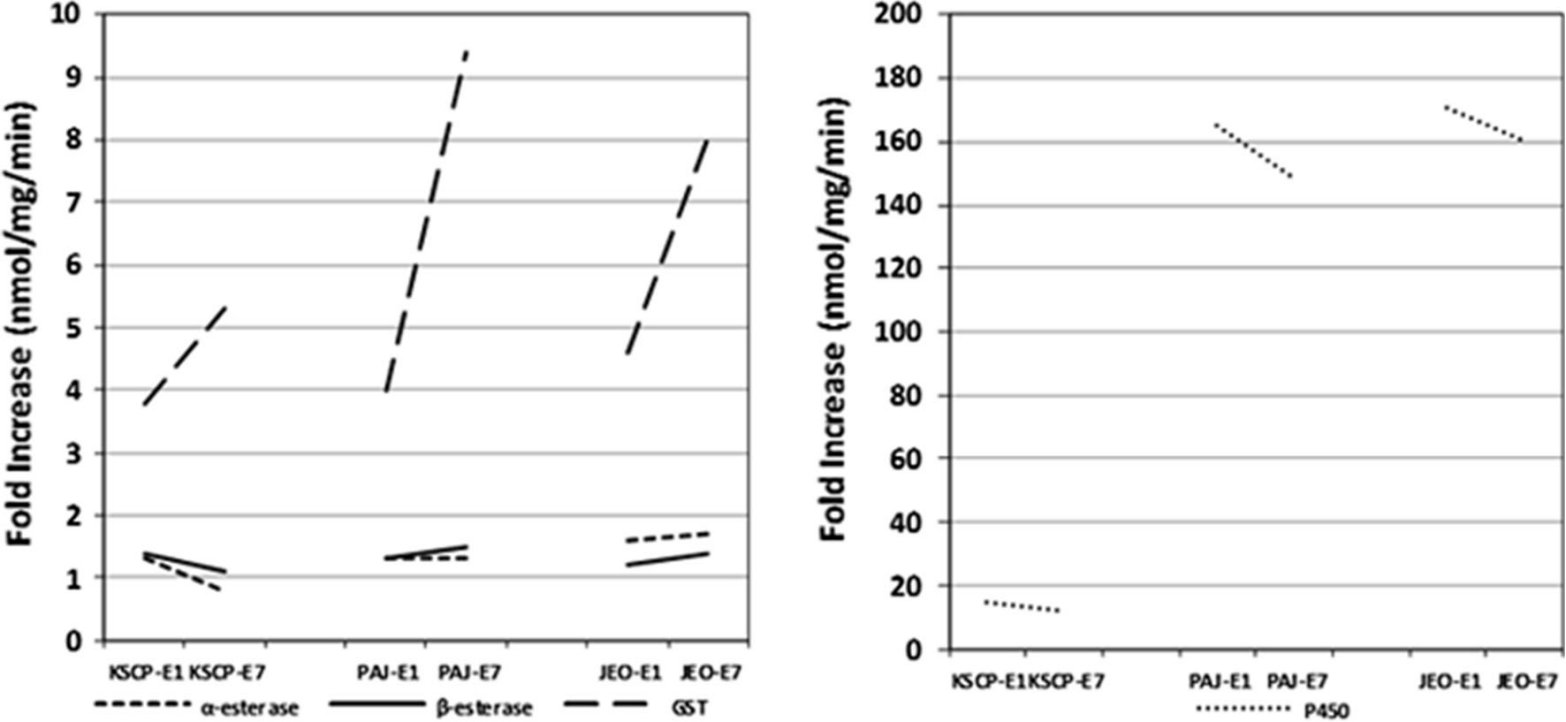
Fold increases/decreases in α-esterase, β-esterase, GST, and P450 enzyme activity for KSCP, PAJ, and JEO strains blood-fed directly on a cow and assayed 1 and 7 days post-blood feeding

For the field strains, P450 quantities for PAJ-U7 and JEO-U7 were 58.8- and 72.8-fold higher when compared to a susceptible colony (KSCP-U7), while quantities of GST, α- and β-esterase increased by 17.1–30.6-, 16.8–18.4-, and 7.8–9.8-fold. The quantity of β-esterase (nmol/mg/min), α-esterase, GST, and P450 ranged from 7.8 to 9.8-, 16.8 to 18.4-, 17.1 to 30.6-, and 58.8 to 72.8-fold higher for PAJ-U7 and JEO-U7 unfed nulliparous females, respectively, when compared to unfed KSCP-U7 females. Similar fold increases were observed in the quantity of β- and α-esterases (range 10.3–13.7-fold) when compared to KSCP-U7 females. PAJ-E1/E7 and JEO-E1/E7 blood-fed females demonstrated much higher levels of GST enzyme activity levels, ranging from 68.2 to 161.1- and from 141.0 to 245.5-fold higher, respectively, when compared to KSCP-U7 females. In addition, there was a 4.0- and 4.6-fold increase in GST activity for PAJ-E1 and JEO-E1, respectively, and 9.4- and 8.2-fold increases observed for PAJ-E7 and JEO-E7, respectively, compared to unfed KSCP-U7 females. P450 demonstrated the highest levels of increased enzyme activity, ranging from 8722.8 to 12,415.8, for PAJ-E1/E7 and JEO-E1/E7 blood-fed females when compared to KSCP-U7 females. When compared to corresponding JEO and PAJ unfed groups, blood-fed females demonstrated 164.9–170.7- and 148.5–160.4-fold increases in enzyme activity for females assayed 1 and 7 days, respectively, after a blood meal (Fig. 6).

## Discussion

Insecticide resistance, defined as >10-fold reduction in sensitivity to insecticides, is a main obstacle for establishing effective chemical control measures against arthropod vectors (Brown and Pal 1971; Munhenga et al. 2008). Most resistance mechanisms can be divided into four groups, behavioral changes (avoidance of insecticide, decreased indoor biting of houses sprayed with insecticides and increased outdoor biting activity) (Lines et al. 1987; Mbogo et al. 1996; Mathenge et al. 2001), cuticular alterations (e.g., thicker or altered cuticles that reduce insecticide penetration (Stone and Brown 1969; Apperson and Georghiou 1975), and metabolic resistance (e.g., rapid breakdown of active ingredients and elimination of insecticides) (Hemingway et al. 2004).

Although increased insecticide resistance might result from various resistance mechanisms, e.g., mutations of target sites and activation of metabolic detoxification enzymes, this paper focuses on the relationship between insecticide resistance and the activation of detoxification enzymes among both unfed and blood-fed groups for a susceptible colony (control) and two field-collected test strains. Metabolic resistance against selected insecticides has been shown to increase in *Cx*. *p*. *pallens* following blood meals by altering the activity of detoxification enzymes rather than target site mutations, which ultimately impact on the selection of available insecticides and implementation of mosquito control strategies (Baldridge and Feyereisen 1986; Sanders et al. 2003). While Baldridge and Feyereisen (1986) observed decreased enzyme activity 4–6 days post-blood feeding when females have completed digestion of the blood meal, this study showed decreased susceptibility/resistance among blood-fed females of all strains against all insecticides tested when compared to unfed females of the same strain. In addition, the activation of all enzymes greatly increased among blood-fed females 1 day post-blood feeding when compared to unfed females of the same strain, and continued to increase among females on day 7 post-blood feeding.

Cytochrome P450-dependent monooxygenases are an important and diverse family of hydrophobic, heme-containing enzymes involved in the metabolism of numerous endogenous and exogenous compounds (Hemingway et al. 2004) and may be involved with the oxidation of insecticides. For example, there are many reports demonstrating elevated P450 monooxygenase activities in insecticide-resistant mosquitoes, frequently in conjunction with altered activities of other enzymes. Vulule et al. (1999) demonstrated elevated oxidase and esterase levels in permethrin-resistant *Anopheles gambiae* from Kenya. Brogdon et al. (1999a, b) reported oxidase-based and esterase-based resistance mechanisms alone and in combination with other enzymes among permethrin-resistant *An. albimanus* from Guatemala. Elevated levels of GST activity have been reported in mosquitoes resistant to organophosphate, organochlorine, and pyrethroid insecticides (Hemingway 2000; Brown 1986; Lumjuan et al. 2005). Nonspecific esterases are documented to play a role in organophosphate resistance and sometimes to pyrethroids (Hemingway et al. 2004; Pathuan et al. 2007). Esterase metabolism contributed to pyrethroid resistance in *An*. *gambiae* (Vulule et al. 1999). and elevation of α-esterase has been correlated to permethrin decreased susceptibility in *Aedes aegypti* (Flores et al. 2005). Insecticide resistance in the PAJ and JEO field populations increased up to 50-fold 1 and 7 days post-blood feeding, while P450 quantities increased up to >100-fold. While P450 may play a major role in the development of insecticide resistance, both before and after blood feeding, the combined increases in enzyme activities of P450, GST, α-esterase, β-esterase may be synergistic, enhancing increased resistance among field populations of *Cx*. *p*. *pallens*.

These results further indicate that the quantity of P450, GST, α-esterase, and β-esterase enzymes increased among both insecticide-susceptible laboratory and field-collected strains 1 and 7 days post-blood feeding, which likely resulted in observed decreased levels of susceptibility to the organophosphate and synthetic pyrethroid insecticides evaluated. Spillings et al. (2008) reported that a fully susceptible *An*. *funestus* strain did not show any significant alteration in susceptibility to insecticides following a blood meal, while increases in decreased susceptibility to insecticides were observed for an insecticide-resistant strain of *An*. *funestus* following a blood meal. This suggests that insecticide detoxification mechanisms involved in insecticide resistance are stimulated by the presence of a blood meal for resistant strains. While this study demonstrated small increased levels of decreased susceptibility/resistance among unfed (KSCP-U7) and the 1 (KSCP-E1) and 7 (KSCP-E7) days post-blood feeding groups, high levels of decreased susceptibility/resistance were observed between the unfed PAJ/JEO-U7) and the 1 (PAJ/JEO-E1) and 7 (PAJ/JEO-E7) days post-blood feeding groups.

Baldridge and Feyereisen (1986) reported upregulation of cytochromes P450 in response in *Cx*. *pipiens* after a blood meal, whereas P450 levels did not change between days 1 and 12 after adult emergence in non-blood-fed mosquitoes, except for a small peak on day 2. However, P450 activity in mosquitoes increased to a plateau that was maintained for 2–4 days after a blood meal and then decreased after day 6. In our results, activation of P450 enzyme activity was the highest in both the susceptible laboratory and resistant field strains after blood feeding, a pattern similar to results of Baldridge and Feyereisen (1986). In the susceptible and resistant field strains, the quantity of P450 increased on day 1 after a blood meal, but decreased by day 7 after a blood meal. In both the susceptible and resistant field strains, increases of α-esterase and β-esterase were ≤1.5-fold, while increases of GST were ≤9.4-fold on days 1 and 7 post-blood feeding. These results indicate that significant increases in P450 enzyme activity in resistant field populations following a blood meal led to increased resistance to organophosphates. However, although the quantity of P450 decreased in field populations by day 7 after a blood meal, resistance to pyrethroids increased. For the susceptible laboratory strain, both insecticide resistance to pyrethroids and the quantity of P450 decreased on day 7 from levels observed on day 1 post-blood feeding. These results indicate that other insecticide-resistant mechanisms affected increased insecticide resistance to pyrethroids, e.g., target site mutation or increased activation of P450 that is regulated by the CYP6D1 gene. In most cases where a link between insecticide resistance and elevated P450 activity has been shown, the *Cyp* gene belonging to the *Cyp*6 family, has been involved. For example, the CYP6D1 gene is responsible for increased pyrethroid resistance in *Musca domestica* due to upregulated transcription (Anderson et al. 1994; Kasai and Scott 2000) and is similarly associated with organophosphate resistance (Anderson et al. 1994; Sabourault et al. 2001).

In the laboratory test, although insecticidal activity to mosquitoes is strongly affected according to nutrition status (Kreß et al. 2014), an evaluation of insecticide susceptibility or resistance of mosquitoes might be conducted using engorged female mosquitoes to obtain more accurate results. These data should be informative on the roles of a blood meal on resistance increase factors of mosquitoes and provide important implications for effective vector control in the Republic of Korea.

Here, our results showed that a synergic effect of four metabolic enzymes in field populations of *Cx*. *pipiens* with increased insecticide resistance, but only P450 might play a major role in the increase of insecticidal resistance after a blood meal because activation of three other resistance-related metabolic enzymes was very low at <10-fold and the sucking of a blood meal is not related to the mutation of target sites such as the *Kdr* or AchE genes. Further studies suppressing activation of the genes related to P450 after sucking a blood meal should be carried out. Our findings demonstrate that suppressing the activation of the P450 protein is a critical implementation for increasing the susceptibility of pesticides and might support critical information on biogenetical control of *Cx*. *pipiens* by gene mutation suppressing the activation of the P450 protein for effective mosquito management in ROK.

## Author contributions

KSC conceived and designed this study; KSC, HCK and TAK wrote the manuscript and KSC, performed the fieldwork and analyzed all data; YRJ supervised this research.
